# Concentration of Healthcare Resources in China: The Spatial–Temporal Evolution and Its Spatial Drivers

**DOI:** 10.3390/ijerph16234606

**Published:** 2019-11-20

**Authors:** Qingbin Guo, Kang Luo

**Affiliations:** 1 School of Economics, Hainan University, Haikou 570228, China; 994076@hainanu.edu.cn; 2 School of Business, Hubei University, Wuhan 430062, China

**Keywords:** concentration of healthcare resources, spatial driving mechanisms, spatial Durbin model

## Abstract

This paper estimated and evaluated the spatial–temporal evolution of the concentration of healthcare resources (HCRs), in 31 provinces in China between 2004 and 2017, by using the entropy method. The spatial Durbin model (SDM) was used to further analyze the mechanisms behind the spatial driving forces at the national and regional levels. The findings revealed that: (i) The concentration of HCRs differed significantly among eastern, central, and western regions. The eastern, followed by the central region, had the highest concentration. Going east to west, the concentration of HCRs in the first echelon decreased, while it increased in the second and third echelons; (ii) places with higher concentrations clustered, while those with lower concentrations agglomerated; and (iii) economic development, population size, and urbanization promoted concentration. Education facilitated HCR concentration in the eastern and central regions, income stimulated HCR concentration in the eastern and western regions, and fiscal expenditure on healthcare promoted HCR concentration in the eastern region. Economic development inhibited HCR concentration in neighboring regions, population size restrained HCR concentration in neighboring areas in the western region, urbanization and income curbed HCR concentration in neighboring areas in the eastern and western regions, and fiscal expenditure on healthcare hindered HCR concentration in neighboring areas in the eastern region. Policy recommendations were proposed toward optimizing allocation of healthcare resources, increasing support for healthcare and education, and accelerating urbanization.

## 1. Introduction

According to the World Health Organization (WHO), everyone has the right to access basic healthcare services, in other words, healthcare accessibility is a universal right. The access of healthcare resources (HCRs) is affected by the government’s supply of people and infrastructure in the HCR field. An imbalance in the spatial distribution of HCRs leads to huge differences in the access of HCRs among residents in different regions. Since the implementation of the new healthcare reforms, China’s healthcare system has significantly improved. However, due to the combined influence of population size, economic conditions, and spatial distance of HCRs [1], HCR concentration remains imbalanced across provinces and cities, gravely affecting the efficiency and benefits of utilizing HCRs [2] and increasing the gap in the health levels of residents. The optimal agglomeration of HCRs is an important measure to improve the utilization efficiency of HCRs and promote the residents to enjoy and maximize the use of HCRs [3]. Simultaneously, in recent years, health insurance policy coverage of medical treatment received outside of individuals’ registered residences has created gaps in the utilization of HCRs and pulled out the countless ties between provinces and cities. Therefore, reexamining HCR concentration and the corresponding spatial driving forces has practical significance for the allocation and utilization of HCRs. A better understanding of HCR concentration patterns is important to achieve health and prosperity among all. It also lays down a solid foundation in healthcare for the achievement of the “two centennial goals”. 

HCR concentration reflects an area’s ability to attract various healthcare elements from neighboring areas or even the entire state to agglomerate locally, thereby generating added value and spillover effects. HCRs mainly include healthcare-related human, material, and financial resources. The main measurement indicators of HCR concentration are the number of medical and healthcare institutions, hospital beds, and medical practitioners in a given area. Relative to residents’ unlimited needs for healthcare services, the HCRs of a state, province, or even a given area are always limited. Therefore, with the limited total amount of HCRs that are available, it is very important and urgent to know how to allocate HCRs reasonably and efficiently. Therefore, the study of the spatial–temporal evolution and its spatial drivers of HCRs can clarify the interaction path of the HCRs among provinces and cities, and then, it can pertinently refine the policy to improve the utilization efficiency of HCRs in China. The study of HCR concentration in China began later than in foreign countries; however, its development has been relatively swift. In particular, with the deepening of healthcare reforms, exploring HCR concentration and the mechanisms behind its spatial driving forces from a microscopic perspective has become a research interest. In terms of area of study, existing studies in HCR concentration have approached the subject from a national, cross-provincial, or provincial level, performing analysis on national data [4,5,6], the data of 31 provinces in China [7], or the data of a given province/city [8,9]; studies have also approached the subject from certain specific areas [10]. In terms of types of research data, scholars have used panel data [11], cross-sectional data [12], and time series data [13]. In terms of research methods, studies largely adopted statistical models (such as the logit model) for analysis [14], and a few studies adopted the questionnaire survey method [15,16], entropy weight and technique for order of preference by similarity to ideal solution (TOPSIS) methods [17], and indicator metrics [18].

The introduction of spatial factors into the study of HCR concentration is conducive to understanding the subject from a more realistic perspective. In recent years, scholars have introduced spatial analysis methods into healthcare research and achieved a range of results [19,20,21]; however, there are a few studies in the literature that used such methods to study the mutual influence of HCRs among provinces and cities. The understanding of spatial driving forces of HCR concentration in each area and their corresponding mechanisms is conducive to optimizing overall HCR concentration in China, minimizing insufficient utilization of HCR caused by imbalanced distribution, and improving HCR concentration and public health. Currently, domestic and foreign scholars have adopted spatial econometric models to quantitatively evaluate mechanisms behind the spatial driving forces of various observations. However, such an approach was rarely adopted in healthcare-related studies. The few studies that introduced spatial analysis techniques were inclined to apply them from a methodological perspective [22]. Therefore, this study employed the entropy weight method to measure the HCR concentration of 31 provinces in China from 2004 to 2017; a spatial weight adjacency matrix based on the obtained data was established and Moran’s *I* was used to examine spatial autocorrelation of the model; and the spatial Durbin model (SDM) was then applied to analyze mechanisms behind the spatial driving forces of HCR concentration in China.

## 2. Methodology

### 2.1. Measuring HCR Concentration in China

HCRs include healthcare-related human, material, and financial resources. Referring to relevant literature and expert opinions, a comprehensive evaluation indicator system was constructed to measure the HCR concentration of provinces and cities, and the entropy method was used to determine the weight of each indicator. The entropy method mainly determines the objective weight according to index variability. Generally speaking, the smaller the information entropy of an index, the greater the degree of variation of the value of the index; and the larger the amount of information provided, the greater the role played in the comprehensive evaluation index system and the greater the weight. On the contrary, the bigger the information entropy of an index, the smaller the variation degree of the index value; and the less the amount of information provided, the smaller the role played in the evaluation system of the comprehensive index and the smaller the weight. To avoid the influence of inconsistent units of variables on the weight determination process, min–max normalization was employed to normalize data and convert them into numerical data with values between 0 and 1. The evaluation indicator system of HCR concentration and the corresponding weights are shown in Table 1.

### 2.2. Verification of Spatial Correlation

The existence of spatial autocorrelation is the premise for conducting a regression analysis using spatial econometric models. Spatial autocorrelation is usually measured by Moran’s *I*. In this study, Moran’s *I* showed the degree of attribute similarity between spatially adjacent areas in terms of HCR concentration. The global spatial autocorrelation of the variable can be expressed as follows:

(1) $$M\mathrm{oran}’s\;I\;=\;\frac{\sum_{i=1}^{n}\sum_{j=1}^{n}W_{ij}(\theta_{i}-\bar{\theta })(\theta_{j}-\bar{\theta })}{B^{2}\sum_{i=1}^{n}\sum_{j=1}^{n}W_{ij}},$$

where $B^{2}\;=\;\frac{1}{n}\sum_{i=1}^{n}{\left(\theta_{i}\;-\;\bar{\theta }\right)}^{2},\bar{\theta }\;=\;\frac{1}{n}\sum_{i=1}^{n}\theta_{i}$; *θ_i_* and *θ_j_* are the HCR concentrations of provinces *i* and *j*, respectively; *n* represents the total number of provinces and cities; and *W_ij_* symbolizes the spatial weight adjacency matrix of attributes of provinces within samples. Generally, the value of *W_ij_* is either 0 or 1, “0” means that the attribute values of provinces are not associated, and “1” indicates that attribute values are closely associated and highly similar. 

### 2.3. Types of Spatial Econometric Models and Model Construction 

Traditional econometric modeling does not consider errors caused by spatial effects on the estimation results. In 1970, Tobler proposed the “first law of geography,” pointing out the existence of geographical dependence and correlation between observations. Spatial econometric models can thus describe the geographical dependence between observations. Some commonly used spatial econometric models for analyzing mechanisms behind spatial drivers include the spatial autoregressive (SAR) model, spatial error model (SEM), and spatial Durbin model (SDM).

(1) Spatial Autoregressive Model (SAR)

(2) $$Y\;=\;\rho WY\;+\;\alpha l_{N}\;+\;X\beta \;+\;\varepsilon ,$$

where *ρ*, *W*, *Y*, *X*, *β*, *αl_N_*, and *ε* represent the spatial autoregressive coefficient, interprovincial N×N spatial weight adjacency matrix, HCR concentration, explanatory variables, N×1 constant term, and random disturbances, respectively.

(2) Spatial Error Model (SEM)

(3) $$Y\;=\;\alpha l_{N}\;+\;X\beta \;+\;u,$$

(4) u = λWu + ε,

where *λ* and *u* symbolize the spatial error coefficient of N×1 HCR concentration and the spatial error (disturbance) term when the independent distribution was respected, respectively; and the meanings of the remaining symbols are consistent with those in the previous formula.

(3) Spatial Durbin Model (SDM)

(5) $$Y\;=\;\rho WY\;+\;\alpha l_{N}\;+\;X\beta \;+\;WX\theta \;+\;\varepsilon ,$$

where θ is the spatial interaction term of the explanatory variable and HCR concentration and the meanings of the remaining symbols are consistent with those in previous formulas. 

When there are spatial lags in the model, the regression results illustrate more than the effect of explanatory variables on HCR concentration. LeSage and Pace [23] suggested that the overall effect should be further divided into direct and indirect effects in order to describe spatial interactions between variables. The direct effect was used to represent the average impact of explanatory variables on corresponding areas. The indirect effect was used to represent the average impact of explanatory variables on the neighboring areas. The overall effect was used to represent the average impact of explanatory variables on all provinces within the sample. The SDM can be rewritten as follows:

(6) $$(I-\rho W)\;y\;=\;X\beta \;+\;\alpha l_{N}\;+\;WX\theta \;+\;\varepsilon ,$$

Equation (6) can be further converted as follows:

(7) $$y\;=\;\sum_{r=1}^{k}S_{r}(W)x_{r}\;+\;V(W)l_{N}\alpha \;+\;V(W)\varepsilon ,$$

where $V(W)\;=\;{\left(I-\rho W\right)}^{-1}\;=\;I\;+\;\rho W\;+\;\rho^{2}W^{2}\;+\;\rho^{3}W^{3}\;+\;\ldots$, $S_{r}(W)\;=\;V(W)(I\beta_{r}\;+\;W\theta_{r})$.

Equation (7) can be converted into a matrix as follows:

(8) $$\left(\begin{array}{l} y_{1}\\ y_{2}\\ \vdots \\ y_{N} \end{array}\right)\;=\;\sum_{r=1}^{k}\left(\begin{array}{l} S_{r}{\left(W\right)}_{11}S_{r}{\left(W\right)}_{12}\ldots S_{r}{\left(W\right)}_{1N}\\ S_{r}{\left(W\right)}_{21}S_{r}{\left(W\right)}_{22}\ldots S_{r}{\left(W\right)}_{2N}\\ \vdots \cdots \cdots \cdots \cdots \cdots \cdots \cdots \cdots \cdots \vdots \\ S_{r}{\left(W\right)}_{N1}S_{r}{\left(W\right)}_{N2}\ldots S_{r}{\left(W\right)}_{N*N} \end{array}\right)\left(\begin{array}{l} x_{1r}\\ x_{2r}\\ \vdots \\ x_{Nr} \end{array}\right)\;+\;V(W)l_{N}\alpha \;+\;V(W)\varepsilon .$$

*Sr(W)_ij_* symbolizes the *i*th and *j*th elements in *Sr(W)*, and *V(W)_i_* represents the *i*th row of *V(W)*.

(9) $$y_{i}\;=\;\sum_{i=1}^{k}\left[S_{r}{\left(W\right)}_{i1}\;+\;S_{r}{\left(W\right)}_{i2}x_{2r}\;+\;\ldots \;+\;S_{r}{\left(W\right)}_{iN}x_{Nr}\right]\;+\;V{\left(W\right)}_{i}l_{N}\alpha \;+\;V{\left(W\right)}_{i}\varepsilon ,$$

The direct effect is equal to the mean of the diagonal elements, *Sr(W)_ii_*; the indirect effect is equal to the mean of the non-diagonal elements, *Sr(W)ij*; and the overall effect is equal to the mean of the sum of the matrices, *Sr(W)*. 

### 2.4. Variable Selection

In this study, the following factors were considered to be spatial driving forces for HCR concentration: (i) economic development—measured using regional Gros Domestic Product(GDP), (ii) population size—measured using the total population at the end of a given year, (iii) urbanization—measured using the proportion of urban land area to total land area, (iv) education—measured using the number of students in colleges and universities in a given region, (v) annual salary—measured using the average annual salary of employees in the healthcare industry, and (vi) fiscal expenditure on healthcare—measured using fiscal expenditure on the healthcare industry. The names, operational definition, and descriptive statistics of the variables are shown in Table 2.

### 2.5. Data Source

Data were extracted from the China Public Health Statistical Yearbook, China Health and Family Planning Yearbook, and China City Statistical Yearbook for the period between 2005 and 2018. In order to maintain data continuity and stability, data for earlier years were recompiled and merged based on the most recent administrative divisions. 

## 3. Empirical Analysis

### 3.1. The Spatial–Temporal Evolution of HCR Concentration in China

**Figure 1:**
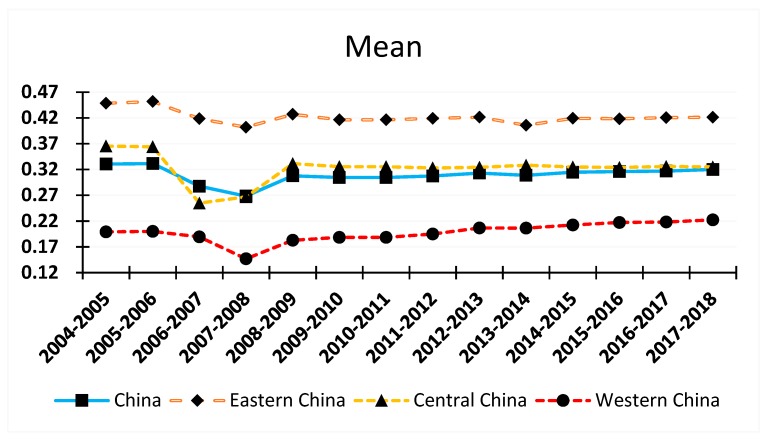
Mean Values of HCR Concentration at National and Regional Levels (Eastern, Central, and Western Regions).

This study used 31 provinces in China as research subjects and employed the entropy method to measure HCR concentration between 2004 and 2017. The average values for HCR concentration at the regional and national levels are presented in Figure 1. As per the findings, trends in changes of regional (eastern, central, and western regions) and national averages of HCR concentration were similar during the period of observation. The HCR concentration at regional and national levels showed a decreasing trend between 2004 and 2007; after increasing drastically in 2008, HCR concentration remained reasonably high thereafter. These changes may be due to the following reasons: In 2004, hospital reforms targeting “commercialization” were implemented, thus HCR concentration increased in 2004. Between 2005 and 2007, the argument that “commercialization is not the correct direction for healthcare reform” emerged, and the government undertook costs of basic public healthcare, thus HCR concentration dropped significantly. With the issuance of “Opinions on Deepening the Reform of the Medical and Health System (Draft for Comment)” by the State Council on 14 October 2008, a new round of healthcare reforms was officially launched. Provinces and municipalities reinforced the reform of the healthcare system and increased financial support from the government. As a result, the situation in medical and health institutions was greatly improved, and HCR concentration in the provinces increased. Across the board, it can be seen that the HCR concentration of provinces in the eastern region was the highest, and greater than that of the national average; the HCR concentration of provinces in the central region did not differ much from the national average, but was slighter higher than the national average; the HCR concentration of provinces in the western region was the lowest, with the concentration level showing a slight increase over time. These findings were consistent with differences in the overall economic development among regions.

Nevertheless, changes in average values for regional HCR concentration were unable to completely reflect the spatial distribution of HCR concentration in China. Hence, sectional data for the corresponding provinces in 2004, 2010, and 2017 were further compared (Table 3). In 2004, 2010, and 2017, provinces belonging to the first echelon (HCR concentration >0.4) accounted for 35.5%, 29.0%, and 25.8% of all provinces and cities, respectively; among these, provinces from the eastern region accounted for 25.8%, 22.6%, and 19.4%, respectively; the proportion of provinces from the central region decreased slightly over time, while the western region remained unchanged at 3.2%. In 2004, 41.9% of provinces belonged to the second echelon (0.2 < HCR concentration <0.4); among these, 6.5% were from the eastern region, 19.4% were from the central region, and 16.1% were from the western region. After 2010, the proportion of second-echelon provinces from the eastern and central regions increased at different levels, while the western region remained at 16.1%. The proportion of provinces that belonged to the third echelon (HCR concentration <0.2) gradually increased with time, as did the proportion of third-echelon provinces and cites from the eastern region; however, the western region remained unchanged at 19.4%. In all the studied years, provinces belonging to the first echelon decreased, while those in the second and third echelons increased. It can thus be concluded that the gap in HCR concentration between the eastern and central regions gradually reduced under the new healthcare reforms and the HCR concentration in the central region showed great potential for further improvement, while it did not change significantly in the western region. These findings could be attributed to factors such as underdeveloped economy, vast empty areas, and low population density in the western region, resulting in the weak targeting of state fiscal expenditure on healthcare, leading to inadequate infrastructure for medical and health institutions and low HCR concentration.

### 3.2. Spatial Correlation Analysis of HCR concentration in China

**Figure 2:**
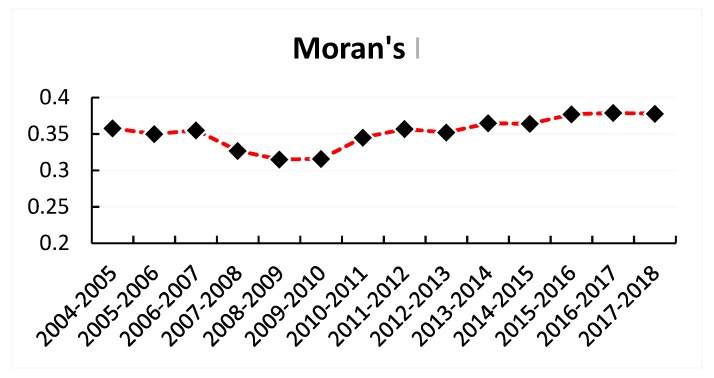
Global Moran’s *I* of HCR Concentration in China.

In the context of regional economic integration, theoretically speaking, there should be a spatial correlation between the HCR concentrations of different provinces and cities. Therefore, the MATLAB2016a software package was used to calculate the global Moran’s *I* of HCR concentration in China from 2004 to 2017 (Figure 2). As presented in Figure 2, the global Moran’s *I* of HCR concentration in China was positive, indicating that the HCR concentration levels between provinces were positively correlated, and suggesting that provinces with a high HCR concentration tended to cluster together, while those with a low HCR concentration agglomerated together. In addition, the chart also demonstrated that spatial correlation of HCR concentration in China experienced a trend in which a decline was followed by an increase. Specifically, there was a noticeable decreasing trend in spatial correlation coefficients from 2007 to 2009. Such a decline could be due to the fact that the years between 2007 and 2009 were the first three years of implementation of the new healthcare reforms, during which reforms were still undergoing an exploratory phase, and provinces were attempting to determine appropriate paths to achieve the national goal given the current situations of their own healthcare systems; hence, the degree of correlation of HCR concentration between provinces declined. Thereafter, with further implementation of reforms, provinces began to exchange accumulated experience and successful practice with each other, which in turn facilitated the increase of the degree of spatial correlation of HCR concentration in China. 

### 3.3. Determining the Spatial Econometric Model for HCR concentration in China

Based on panel data from 2004 to 2017 and referring to the method proposed by LeSage and Pace (2009) [23], the MATLAB2016a software and spatial econometrics software package was used to test data and determine the spatial econometric model suitable for the study. The test for form of spatial dependence (Table 4) showed that the values of Lagrange multiplier lag (LM-lag) and Robust LM-lag were 11.5026 and 29.2864, respectively (*p* < 0.01); and the values of LM-error and Robust LM-error were 186.0900 (*p* < 0.01) and 1.3849 (*p* > 0.01), respectively, indicating that a lagged spatial dependence existed in the panel model of HCR concentration in China. The test results of the spatial econometric models revealed that the *p*-values of spatial lag and spatial error coefficients of LR and Wald were smaller than 0.01, rejecting the zero hypothesis that SDM degenerates into SAR and SEM; hence, there were sufficient reasons to choose SDM as the model to explore the mechanisms behind the spatial driving forces of HCR concentration in China.

Furthermore, results of the Hausman test showed that the fixed effects model should be used. As reported in Table 5, the goodness of fit of the spatiotemporal fixed effects model (0.8512) and its log-likelihood value (520.7912) were greater than those of the random effects model (0.6806, 437.6806), spatial fixed effects model (0.7574, 471.3144), and temporal fixed effects model (0.7535, 487.4108), suggesting that the spatiotemporal fixed effects model was most suitable for this study.

However, the adopted approach tended to cause a bias in estimation. Moreover, direct, indirect, and overall effects should be considered when applying spatial econometric models [24,25]. Therefore, referring to the method used by Lee and Yu [26], error correction was conducted for the regression results of the model; and the overall effect of HCR concentration was decomposed into direct and indirect effects with reference to the practice of Lesage and Pace [23]. The direct effect reflected the impact of changes in the explanatory variables of a given place on its HCR concentration, while the indirect effect reflected the impact of changes in the explanatory variables of a given place on the HCR concentration of neighboring areas (Table 6).

The direct effects of economic development (GDP) on HCR concentration at the regional and national levels were significant and positive, which were consistent with the findings of a scholar (Yu, J.N., 2017) [27], while indirect effects were significant and negative. The overall effects of economic development on national HCR concentration were found to be positive, but not statistically significant. These findings suggested that economic development played a fundamental role in promoting the HCR concentration of a given province (city); however, it had a negative spillover effect on the HCR concentration of neighboring provinces and cities. The reason could be that the residents of regions with a more developed economy were more inclined to have better living conditions and pay more attention to physical health as well as healthcare resources and facilities; therefore, they had higher demands on the local government, which indirectly led to a reinforcement of HCR concentration efforts by the local government. Moreover, since the flow of resources are usually profit-driven, provinces with more developed economies were more likely to attract HCRs from neighboring provinces and cities, leading to the “siphon effect” of economic development on HCR concentration to exceed the “diffusion effect.”

The coefficients of direct effects, indirect effects, and overall effects of population size (Pop) on HCR concentration were large. Specifically, the indirect effects of population size on the HCR concentration of the eastern and central regions were positive, yet not statistically significant; and those on national HCR concentration and the HCR concentration in the western region were significant and negative. The remaining effects of population size were found to be significant and positive. These findings suggested that population size played a significant role in promoting the overall HCR concentration in China and the HCR concentration of a given place, which were roughly consistent with the findings of a scholar (Yu, J.N., 2017) [27]. This could be due to the fact that a larger population size led to an increased demand for HCRs, which increased the pressure on the government to improve HCR concentration, thereby promoting the increase in HCR concentration. The significant and negative indirect effects of population size on national HCR concentration and that of the western region could be due to the vast empty areas, low population density, low economic development, and insufficient transportation facilities in western China, leading to the increase in commuting costs of HCRs. As a result, cities that were more developed, such as provincial capitals, tended to become “low-lying areas” in which HCRs flowed, thereby negatively affecting the HCR concentration of neighboring areas. 

The direct effects, indirect effects, and overall effects of urbanization level (Urb) were significant and positive, with the exception of its indirect effects on national HCR concentration and the HCR concentration of the central region (*p* > 0.1), indicating that urbanization significantly facilitated the concentration of HCRs in a given region in China, which were consistent with the view that the development of urbanization was beneficial to the improvement of HCR concentration (Adolf, W., 1891) [28]. On one hand, the development of urbanization facilitated an increase in the scale and intensity of HCR production in a given area, thereby promoting HCR concentration in the area. On the other hand, the sustained development of urbanization connected originally scattered areas into a single entity and broke down barriers caused by administrative divisions; as a result, provinces were able to share and jointly establish HCRs, creating a positive spillover effect. 

The direct effects of education (Stu) on the overall HCR concentration in China and the HCR concentration of the eastern and central regions were found to be significant and positive. The overall effects were positive at regional and national levels; however, such effects were significant only in the eastern and central regions. The indirect effects were not statistically significant. None of the effects were found to be statistically significant on HCR concentration in provinces of the western region. Students in colleges and universities are the “reserve army” for health and medical personnel and the main pillars for the improvement of HCR concentration. In the eastern and central regions, there are many well-known universities, such as the “985 project” and “211 project” universities, which can directly supply medical and health personnel to various provinces. A majority of the medical students from universities, however, tend to complete their internship at medical and health institutions with strong regional cooperation ties to their schools; hence, the indirect effects of education were small and statistically insignificant. Provinces in the western region have comparatively fewer colleges and students; hence, the effects of education on the improvement of HCR concentration in the region were not prominent. 

The overall effects of salary (Wag) on national HCR concentration and the HCR concentration of the western region and the indirect effects of salary on national HCR concentration and the HCR concentration in the eastern and western regions were significant and negative. The direct effects of salary on HCR concentration at the regional and national levels were significant and positive. These findings highlighted that the increase of annual salary in the medical and healthcare industry promoted HCR concentration in a given province (city), and inhibited HCR concentration in neighboring provinces and cities. Salary increases in the medical and healthcare industry directly stimulated the engagement of doctors, nurses, workers, and management personnel in the industry, as well as attracted corresponding human resources from neighboring provinces and cities, resulting in a negative spillover effect. 

The direct effects of fiscal expenditure on healthcare (Exp) on national HCR concentration and the HCR concentration of the eastern region were significant and positive, which were consistent with the view that fiscal expenditure on healthcare promoted the concentration of HCR concentration (Zhang, X; Gong, S.H., 2014) [29] and the indirect effects of fiscal expenditure on national HCR concentration and the HCR concentration of the eastern region were significant and negative. The remaining effects were statistically insignificant. The reason could be due to the well-developed economy of provinces in the eastern region, with local governments having more financial resources to make larger investments in healthcare, leading to an increase in HCR concentration in the eastern region. However, financial support from the government tended to lead to a certain degree of over-dependence on healthcare institutions; hence, its effects were not substantial. In addition, the significant and negative indirect effects of fiscal expenditure on healthcare at the national level and in the eastern region indicated that the increase in fiscal expenditure on healthcare in a given place tends to cause a crowding-out effect on other provinces and cities, which is not conducive to the improvement of HCR concentration there; competition for fiscal expenditure on healthcare also exists among provinces and cities. 

### 3.4. Robustness Test

In order to verify the reliability of the above analysis results, the robustness of results was tested using a transformation of the spatial weights matrix. Referring to the study by Parent et al. [30], the spatial adjacency matrix was replaced by the geographical distance weight matrix. The Lagrange multiplier (LM), Wald test, likelihood-ratio (LR) test, and Hausman test were adopted and the panel SDM model with spatiotemporal fixed effects was used to test the spatial driving mechanisms behind HCR concentration at regional and national levels. The findings on the influence orientation of the variables and significance of the results were mainly consistent with the above analysis, which confirmed that findings of the present study have satisfactory robustness. Detailed results are presented in Table 7.

## 4. Conclusions and Suggestions

Using the entropy method, this study estimated and evaluated the spatial–temporal evolution of HCR concentration in 31 provinces in China between 2004 and 2017. The SDM was used to further analyze the mechanisms behind the spatial driving forces at the national and regional levels (eastern, central, and western regions). The conclusions are as follows: (i) HCR concentration differed significantly among the eastern, central, and western regions. The provinces in the eastern region had the highest HCR concentration, followed by the central region and the HCR concentration was the lowest in provinces from the western region. In addition, the number of provinces with HCR concentration belonging to the first echelon gradually decreased from east to west, while the number of provinces with HCR concentration belonging to the second and third echelons gradually increased from east to west; (ii) the global Moran’s *I* of HCR concentration in China was positive, indicating that provinces with a higher HCR concentration generally tended to cluster together, while those with a lower HCR concentration tended to agglomerate together in China; and (iii) the mechanisms behind the spatial driving forces of HCR concentration had different degrees of promoting effects on HCR concentration in China depending on the driver variable and region. From a national perspective, economic development, urbanization, education situation, fiscal expenditure on health care, annual salary of the healthcare industry, and population size of a given place were found to share positive relationships with HCR concentration and promote the increase in HCR concentration in that region. However, economic development, population size, annual salary of the healthcare industry, and fiscal expenditure on healthcare were found to curb the increase of HCR concentration in neighboring provinces and cities. In the eastern region, economic development, urbanization, education situation, fiscal expenditure on healthcare, annual salary of the healthcare industry, and population size were found to be the direct driving forces of HCR concentration in provinces within the region. Urbanization in the eastern region was found to have substantial facilitating effects on the ability of neighboring provinces to attract HCRs; however, economic development, annual salary of the healthcare industry, and fiscal expenditure on healthcare were found to have a negative effect on HCR concentration in neighboring provinces and cities. In the central region, economic development, urbanization, education situation, and population size were found to be the direct driving forces of HCR concentration in provinces within the region; however, economic development of a given place tended to hinder HCR concentration in neighboring areas. In the western region, economic development, annual salary of the healthcare industry, and population size promoted HCR concentration in provinces within the region, while significantly inhibiting HCR concentration in neighboring provinces and cities; however, urbanization was found to not only promote HCR concentration of a given area, but also stimulate HCR concentration in neighboring areas. 

In response to the aforementioned conclusions, the following policy recommendations were proposed: (i) Optimize the allocation of healthcare resources. Optimizing limited medical and healthcare resources and improving the overall concentration of such resources in various provinces are global problems. With the growing need for better physical health, the utilization of healthcare resources can only be maximized through rational allocation. At present, the difference of regional HCR concentration in China is very obvious, which is embodied in the eastern region having more HCRs than the central and western regions. In addition, slow economic development and vast unpopulated areas in these regions have led to low coverage and imbalanced distribution of HCRs. Therefore, it is necessary to optimize HCRs between and within regions and provide greater support to the development of the healthcare industry in the western region, so as to achieve the joint establishment and common enjoyment of HCRs; (ii) increase the support for healthcare and education in relevant fields. At present, the average salary of the healthcare industry and fiscal expenditure on the industry have insignificant promoting effects on HCR concentration in the central region. Moreover, the education situation and fiscal expenditure on healthcare also have insignificant promoting effects on HCR concentration in the western region. In addition to acting as the foundation and an important safeguard for the development of the healthcare sector, fiscal expenditure is also an important source of salaries for employees in the medical and healthcare industry. It is only with the support of sufficient funding that a region can be enabled to bring in healthcare personnel, facilities, and equipment to promote the concentration of HCRs in the region. The education situation in the region signifies the potential of reserve forces of the healthcare industry and serves as one of the major components of HCRs. Therefore, in addition to the continued increase of fiscal expenditure on healthcare, the government should also actively support the development of the education industry in the western region, improve the pool of talent, and fundamentally enhance the ability of the region to “create new blood; and (iii) the process of urbanization must be accelerated. Urbanization not only plays a decisive role in the HCR concentration in a given province, but also has significant indirect effects on HCR concentration in neighboring areas. However, urbanization levels in China remain low, and the rural population—which accounts for approximately 40% of the total population—remains a barrier for further improvement in HCR concentration. For this reason, the government should spare no effort in continuing to advance the process of urbanization in order to maximize the vast potential of urbanization in improving HCR concentration.

Although this paper examined the spatial drivers of HCR concentration in China by adding the spatial weight matrix, to a certain extent, it made up for the shortcomings of previous studies, which were limited in expressing the spatial distribution of HCR concentration and ignored its spatial interaction; however, the spatial weight matrix does not have the time sequence, and cannot be consistent with the various HCR data. On the other hand, this paper only studied the spatial–temporal evolution and its spatial drivers of HCR concentration from three regions in eastern China, central China, and western China, and ignored the influence of the huge difference of the administrative area and population factors among provinces and cities on HCR concentration, which will be the focus of future technical improvement and research.

## Figures and Tables

**Table 1:** Evaluation Indicator System of Healthcare Resources Concentration and the Corresponding Weights.

Primal Indicator	Secondary Indicators	Weights
Concentration of Healthcare Resources	Number of Hospitals	0.126
	Number of Community Health Service Centers/Stations	0.175
	Number of Certified Physician Assistants	0.083
	Number of Certified Physicians	0.081
	Number of Registered Nurses	0.079
	Number of Managers in Medical Institutions	0.076
	Number of Workers in Medical Institutions	0.085
	Number of Healthcare Practitioners/1000 People	0.085
	Total Assets of Health Institutions (RMB 1000)	0.093
	Number of Hospital Beds/1000 People	0.118

**Table 2:** Variable and Descriptive Statistics (2004–2017).

Variables	Unit	Indicator	Observations	Mean	Max	Min	SD
Concentration of HCRs	/	/	434	0.309	0.752	0.01	0.171
Economic Development (GDP)	RMB 1 Billion	Regional GDP	434	1562.597	8970.523	22.034	1517.425
Population Size (Pop)	Million	Year-end population	434	43.0715	111.6900	2.7635	27.2375
Urbanization Level (Urb)	%	% of urban land to total land area	434	51.55	89.60	20.85	14.82
Education (Stu)	Number	Number of students in colleges and universities in the region	434	525,563.11	2,015,345.00	55.00	478,440.99
Annual Salary (Wag)	RMB	Average annual salary in the healthcare industry	434	36,225.85	183,362.23	3648.97	20,969.68
Fiscal Expenditure on Healthcare (Exp)	RMB 1 Billion	Fiscal expenditure on health care	434	20.177	130.756	0.435	19.698

**Table 3:** Regional Spatial Distribution of HCR Concentration in 2004, 2010, and 2017.

Data Range Region	China			Eastern China			Central China			Western China		
	2004	2010	2017	2004	2010	2017	2004	2010	2017	2004	2010	2017
>0.4	35.5	29.0	25.8	25.8	22.6	19.4	6.5	3.2	3.2	3.2	3.2	3.2
0.2–0.4	41.9	45.2	48.4	6.5	9.7	9.7	19.4	19.4	22.6	16.1	16.1	16.1
<0.2	22.6	25.8	25.8	3.2	3.2	6.5	0	3.2	0	19.4	19.4	19.4

Analysis of spatial driving mechanisms of HCR concentration in China; numerical values in year columns denote percentages.

**Table 4:** Results of the Spatial Econometric Model Test.

Objectives	Methods	Results	*p*-value
Form of Spatial Dependence (lag, error, or both)	LM test no spatial lag	11.5026 ***	0.001
	Robust LM test no spatial lag	29.2864 ***	0.000
	LM test no spatial error	186.0900 ***	0.000
	Robust LM test no spatial error	1.3849	0.680
Selection of Spatial Econometric Model (SAR, SEM, and SDM)	Wald spatial lag	203.8738 ***	0.000
	LR spatial lag	136.0359 ***	0.000
	Wald spatial error	220.1195 ***	0.000
	LR spatial error	354.2586 ***	0.000

Remark: * *p* < 0.1, ** *p* < 0.05, *** *p* < 0.01. The same remark applies to all of the following tables.

**Table 5:** Estimation Results of Fixed Effect by Spatial Durbin Model (SDM).

Statistics	Random Effect	Spatial Fixed Effect	Temporal Fixed Effect	Spatiotemporal Fixed Effects
R^2^	0.6806	0.7574	0.7535	0.8512
Log-likelihood	437.6806	471.3144	487.4108	520.7912
Observation	434	434	434	434

**Table 6:** Direct, Indirect, and Overall Effects of Factors Affecting HCR concentration in China.

Variable		China	Eastern China	Central China	Western China
**Direct Effect**	GDP	0.000002 *** (4.339)	0.000003 ** (2.427)	0.000007 ** (2.047)	0.000007 *** (5.777)
	Pop	0.000037 *** (11.220)	0.000027 *** (3.417)	0.000034 ** (2.411)	0.000034 *** (8.809)
	Urb	0.004388 *** (9.049)	0.001679 * (1.669)	0.009106 ** (2.296)	0.001228 *** (2.695)
	Stu	0.000000 * (1.881)	0.000000 * (1.910)	0.000000 * (1.678)	0.000000 (1.604)
	Wag	0.000002 *** (3.488)	0.000007 *** (4.401)	0.000003 (0.732)	0.000001 ** (2.082)
	Exp	0.000000 *** (3.606)	0.000000 *** (3.865)	−0.000000 (−0.392)	0.000000 (0.222)
Indirect Effect	GDP	−0.000002 ** (−2.152)	−0.000004 *** (−2.730)	−0.000009 *** (−2.601)	−0.000011 *** (−6.824)
	Pop	−0.000012 ** (−2.156)	0.000015 (1.473)	0.000001 (0.039)	−0.000020 *** (3.607)
	Urb	0.000152 (0.203)	0.002032 * (1.673)	−0.005185 (−1.303)	0.003092 *** (4.878)
	Stu	−0.000000 (−1.103)	0.000000 (0.191)	0.000000 (0.435)	−0.000000 (−0.318)
	Wag	−0.000004 *** (−3.640)	−0.000008 *** (3.880)	0.000003 (0.696)	−0.000002 *** (−2.754)
	Exp	−0.000000 ** (−2.486)	−0.000000*** (−2.730)	0.000000 (0.183)	0.000000 (0.068)
Overall Effect	GDP	0.000001 (0.855)	−0.000001 (−0.927)	−0.000002** (−2.027)	−0.000004 *** (−3.354)
	Pop	0.000025 *** (5.035)	0.000043 *** (6.075)	0.000035 *** (8.568)	0.000014 *** (3.142)
	Urb	0.004540 *** (8.999)	0.003711 *** (5.464)	0.003921 *** (7.468)	0.004320*** (9.868)
	Stu	0.000000 (0.577)	0.000000* (1.945)	0.000000 * (1.860)	−0.000000 (−0.895)
	Wag	−0.000001 * (1.949)	−0.000001(−0.669)	0.000000 (0.040)	−0.000001 * (−1.747)
	Exp	−0.000000 (−0.319)	0.000000 (0.237)	−0.000000 (−0.586)	0.000000 (0.279)

Note: Figures in brackets are *t*-values.*, **, *** mean that the statistics were significant at the statistical significance levels of 1,5,10 percent. GDP is the economic development, Pop is the population size, Urb is the urbanization level, Stu is the education, Wag is the annual salary, Exp is the fiscal expenditure on healthcare.

**Table 7:** Direct, Indirect, and Overall Effects of the Model Based on Geographic Distance Weight Matrix.

Variables		China	Eastern China	Central China	Western China
**Direct Effect**	GDP	0.000002 *** (4.554)	0.000002 * (1.900)	0.000006 ** (2.349)	0.000006 *** (6.743)
	Pop	0.000032 *** (14.189)	0.000022 *** (3.978)	0.000029 ** (2.500)	0.000030 *** (8.023)
	Urb	0.003965 *** (10.057)	0.001408 * (1.805)	0.006763 ** (2.348)	0.002035 *** (2.709)
	Stu	0.000000 * (1.775)	0.000000 * (1.952)	0.000000 (1.605)	0.000000 (1.357)
	Wag	0.000002 *** (4.987)	0.000006 *** (5.237)	0.000003 (0.895)	0.000001 ** (2.226)
	Exp	0.000000 *** (3.025)	0.000000 *** (4.345)	−0.000000 (−0.674)	0.000000 (0.451)
Indirect Effect	GDP	−0.000002 ** (−2.235)	−0.000003 *** (−3.793)	−0.000007 *** (−2.987)	−0.000009 *** (−9.856)
	Pop	−0.000010 ** (−2.038)	0.000012 (1.008)	0.000001 (0.821)	−0.000017 *** (−3.998)
	Urb	0.000137 (0.508)	0.001907 (1.409)	−0.004925 (−1.465)	0.002645 *** (5.458)
	Stu	−0.000000 (−0.934)	0.000000 (0.803)	0.000000 (0.653)	−0.000000 (−0.985)
	Wag	−0.000005 *** (−4.578)	−0.000007*** (−4.936)	0.000003 (0.803)	−0.000002 *** (−3.783)
	Exp	−0.000000 ** (−2.079)	−0.000000 *** (−3.785)	0.000000 (0.907)	0.000000 (0.708)
Overall Effect	GDP	0.000001 (0.923)	−0.000000 (−1.005)	−0.000001 ** (−2.154)	−0.000003 *** (−3.674)
	Pop	0.000023 *** (5.872)	0.000037 *** (9.008)	0.000029 *** (9.654)	0.000010 *** (2.985)
	Urb	0.003908 *** (9.356)	0.002901 *** (6.459)	0.004528 *** (8.485)	0.003958 *** (7.592)
	Stu	0.000000 (0.782)	0.000000 * (1.788)	0.000000 * (1.900)	−0.000000 (−0.706)
	Wag	−0.000001 * (−1.687)	−0.000001 (−0.892)	0.000000 (0.140)	−0.000001 * (−1.876)
	Exp	−0.000000 (−0.875)	0.000000 (0.450)	−0.000000 (−0.765)	0.000000 (0.682)

Note: Figures in brackets are *t*-values.*, **, *** mean that the statistics were significant at the statistical significance levels of 1,5,10 percent. GDP is the economic development, Pop is the population size, Urb is the urbanization level, Stu is the education, Wag is the annual salary, Exp is the fiscal expenditure on healthcare.
